# Supplementary material for: Artificial Fluorescent Glucosinolates (F-GSLs) Are Transported by the Glucosinolate Transporters GTR1/2/3
Source: Int J Mol Sci. 2023 Jan 4;24(2):920. doi: 10.3390/ijms24020920 (PMC9862856; doi:10.3390/ijms24020920)
Supplement: Supplementary file 1 [file ijms-24-00920-s001.zip › ANOVA tables.pdf]

Figure 2 ANOVA table

Model: F-GSL~RNA-expressed

F-GSL	Sum of Squares	P Value	
GSL-A-BODIPY	457.2	4.92e-07	***
GSL-A-Dansylamide	755663	<2e-16	***
GSL-A-Rhodamine	0.39	0.757	
GSL-A-Fluorescein	16.45	3.15e-12	***
GSL-B-DNS	663299	<2e-16	***
GSL-B-Fluorescein	2161.2	<2e-16	***
GSL-B-NBD	225010	<2e-16	***

Signif. codes: 0 '***' 0.001 '**' 0.01 '*' 0.05 '.' 0.1 ' ' 1

Figure 3 ANOVA table

Model: F-GSL~RNA-expressed

F-GSL	Sum of Squares	P Value	
GSL-A-Dansylamide	359018	<2e-16	***
GSL-A-Fluorescein	6.692	8.46e-09	***
GSL-B-DNS	500235	<2e-16	***
GSL-B-Fluorescein	922.1	4.23e-07	***
GSL-B-NBD	226027	<2e-16	***

Signif. codes: 0 '***' 0.001 '**' 0.01 '*' 0.05 '.' 0.1 ' ' 1
