# Supplementary material for: Artificial Fluorescent Glucosinolates (F-GSLs) Are Transported by the Glucosinolate Transporters GTR1/2/3
Source: Int J Mol Sci. 2023 Jan 4;24(2):920. doi: 10.3390/ijms24020920 (PMC9862856; doi:10.3390/ijms24020920)
Supplement: Supplementary file 1 [file ijms-24-00920-s001.zip › NMR_rawdata_GSL-A_Bodipy_Rhodamine_Dansylamide.zip!/NMR raw data PK/GSL-A-Dansylamide/1/pdata/1/email_jic145270_oa_1_1.pdf]

TU Braunschweig NMR-Labor d. Chem. Institute
Kunde Jimidar
Substanz-Code KL-CJ4-007
Exp. 1H
protonpp.ibk_35 MeOD {D:\u} nmr 4

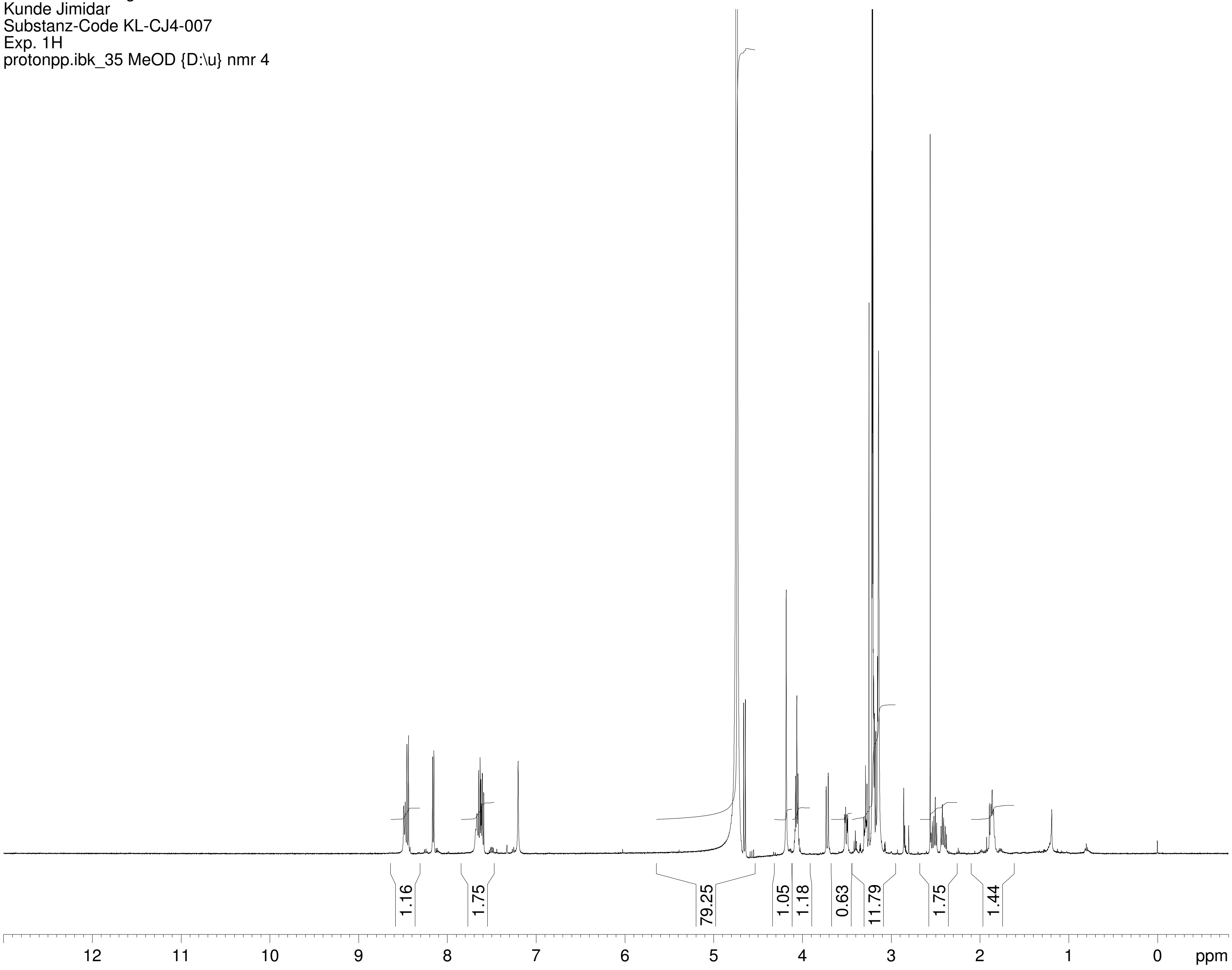

F1P: 13 ppm, F2P: -0.8 ppm

Scale: 0.6000 ppm/cm, 300.2 Hz/cm

Current Data Parameters

NAME	jic145270_oa
EXPNO	1
PROCNO	1

F2 - Acquisition Parameters

Date_	20210906
Time	22.59 h
INSTRUM	AVIIIHD500
PROBHD	Z125869_0056 (
PULPROG	zg30
TD	65536
SOLVENT	MeOD
NS	24
DS	2
SWH	10000.000 Hz
FIDRES	0.305176 Hz
AQ	3.2767999 sec
RG	45.76
DW	50.000 usec
DE	10.00 usec
TE	298.0 K
D1	2.00000000 sec
TD0	1
SFO1	500.3230895 MHz
NUC1	1H
P0	3.33 usec
P1	10.00 usec
PLW1	18.00000000 W

F2 - Processing parameters

SI	32768
SF	500.3200593 MHz
WDW	EM
SSB	0
LB	0 Hz
GB	0
PC	1.00
SR	59.32 Hz
HZpPT	0.305176 Hz
