# Supplementary material for: Artificial Fluorescent Glucosinolates (F-GSLs) Are Transported by the Glucosinolate Transporters GTR1/2/3
Source: Int J Mol Sci. 2023 Jan 4;24(2):920. doi: 10.3390/ijms24020920 (PMC9862856; doi:10.3390/ijms24020920)
Supplement: Supplementary file 1 [file ijms-24-00920-s001.zip › NMR_rawdata_GSL-A_Bodipy_Rhodamine_Dansylamide.zip!/NMR raw data PK/GSL-A-Dansylamide/2/pdata/1/email_jic145270_oa_2_1.pdf]

TU Braunschweig NMR-Labor d. Chem. Institute
Kunde Jimidar
Substanz-Code KL-CJ4-007
Exp. 13C-CPD
c13cpd.ibk_35 MeOD {D:\u} nmr 4

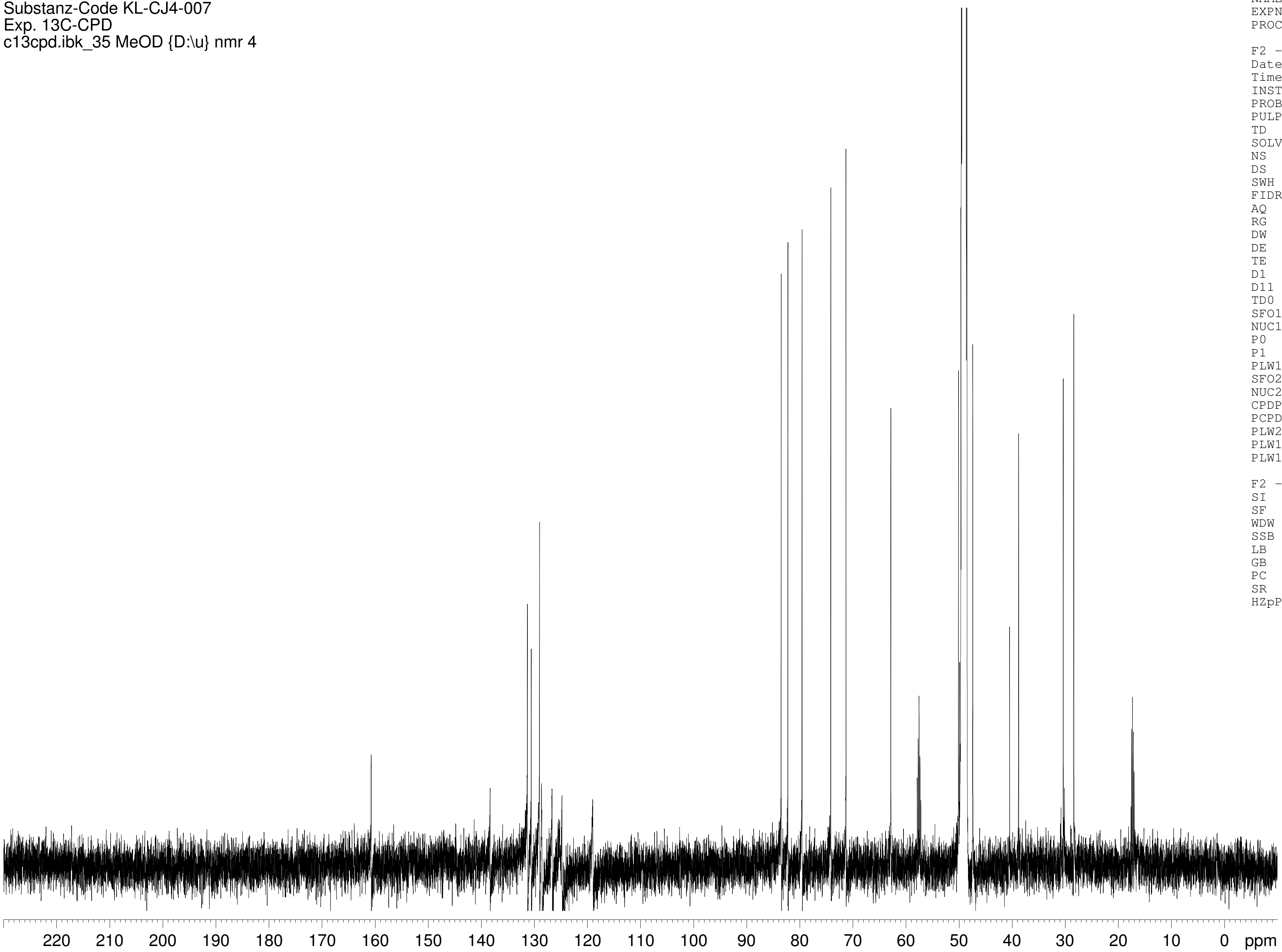

F1P: 230 ppm, F2P: -10 ppm
Scale: 10.00 ppm/cm, 1258 Hz/cm

Current Data Parameters

NAME	jic145270_oa
EXPNO	2
PROCNO	1

F2 - Acquisition Parameters

Date_	20210907	
Time	4.11	h
INSTRUM	AVIIIHD500	
PROBHD	Z125869_0056 (	
PULPROG	zgpg30	
TD	131072	
SOLVENT	MeOD	
NS	4096	
DS	4	
SWH	31250.000	Hz
FIDRES	0.476837	Hz
AQ	2.0971520	sec
RG	186.82	
DW	16.000	usec
DE	18.00	usec
TE	298.0	K
D1	2.38000011	sec
D11	0.03000000	sec
TD0	1	
SFO1	125.8196527	MHz
NUC1	13C	
P0	2.83	usec
P1	8.50	usec
PLW1	76.73300171	W
SFO2	500.3220013	MHz
NUC2	1H	
CPDPRG[2	waltz16	
PCPD2	80.00	usec
PLW2	18.00000000	W
PLW12	0.28125000	W
PLW13	0.14147000	W

F2 - Processing parameters

SI	65536	
SF	125.8053858	MHz
WDW	EM	
SSB	0	
LB	1.00	Hz
GB	0	
PC	1.40	
SR	-179.18	Hz
HZpPT	0.476837	Hz
