# Supplementary material for: Artificial Fluorescent Glucosinolates (F-GSLs) Are Transported by the Glucosinolate Transporters GTR1/2/3
Source: Int J Mol Sci. 2023 Jan 4;24(2):920. doi: 10.3390/ijms24020920 (PMC9862856; doi:10.3390/ijms24020920)
Supplement: Supplementary file 1 [file ijms-24-00920-s001.zip › NMR_rawdata_GSL-A_Bodipy_Rhodamine_Dansylamide.zip!/NMR raw data PK/GSL-A-Dansylamide/3/pdata/1/email_jic145270_oa_3_1.pdf]

TU Braunschweig NMR-Labor d. Chem. Institute
Kunde Jimidar
Substanz-Code KL-CJ4-007
Exp. 13C-Dept135
c13dept135sn.ibk_35 MeOD {D:\u} nmr 4

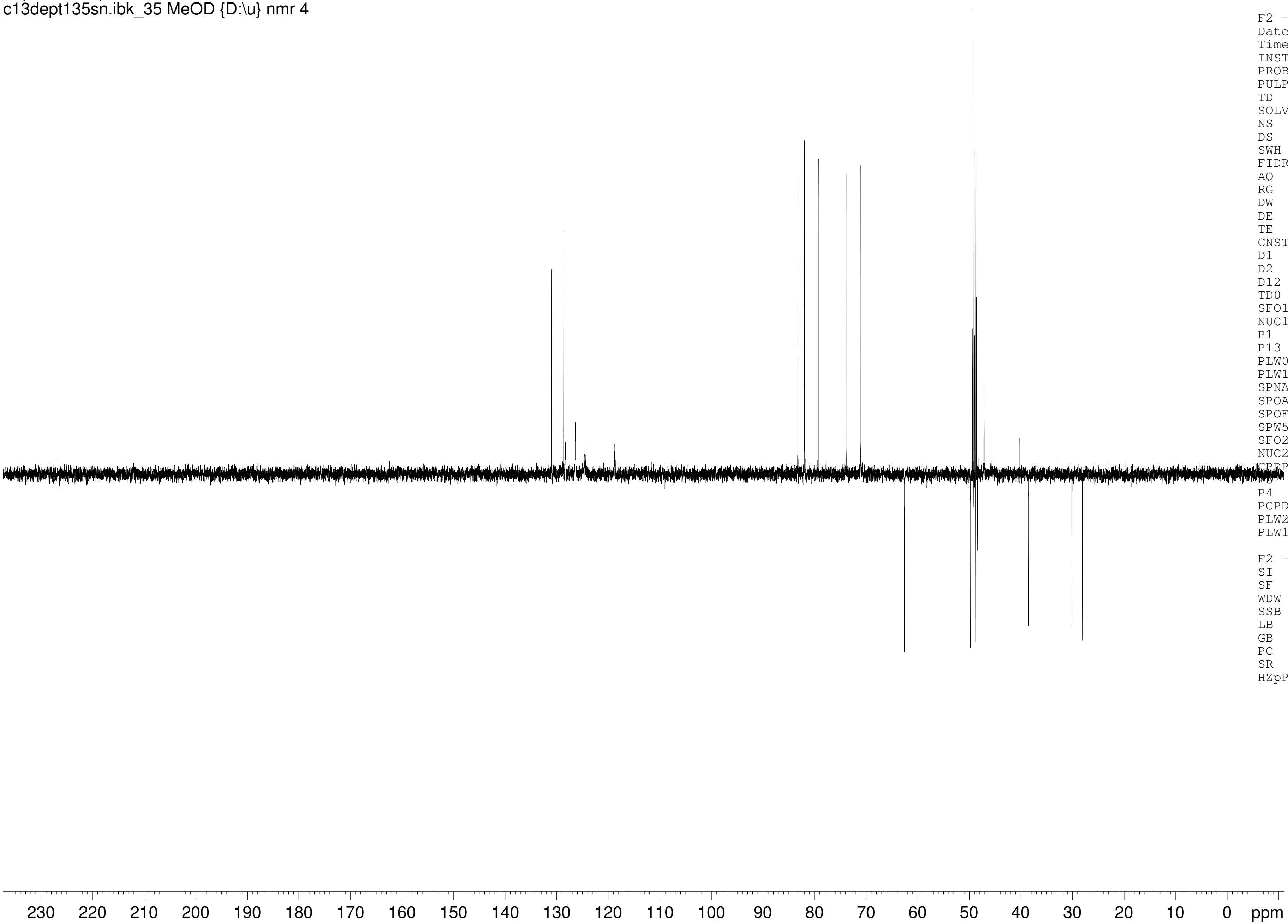

F1P: 240 ppm, F2P: -10 ppm

Scale: 10.35 ppm/cm, 1302 Hz/cm

```
Current Data Parameters
NAME       jic145270_oa
EXPNO                 3
PROCNO                1

F2 - Acquisition Parameters
Date_          20210907
Time               6.32 h
INSTRUM      AVIIIHD500
PROBHD   Z125869_0056 (
PULPROG      deptsp135
TD               131072
SOLVENT            MeOD
NS                 2016
DS                    8
SWH           31250.000 Hz
FIDRES         0.476837 Hz
AQ            2.0971520 sec
RG               186.82
DW               16.000 usec
DE                18.00 usec
TE                298.0 K
CNST2       145.0000000
D1           2.00000000 sec
D2           0.00344828 sec
D12          0.00002000 sec
TD0                  21
SFO1        125.8196527 MHz
NUC1                13C
P1                 8.50 usec
P13             2000.00 usec
PLW0      0 W
PLW1        76.73300171 W
SPNAM[5]    Crp60comp.4
SPOAL5            0.500
SPOFFS5   0 Hz
SPW5         8.47049999 W
SFO2        500.3220013 MHz
NUC2                 1H
CPDPRG[2        waltz16
[illegible]       10.00 usec
P4                20.00 usec
PCPD2             80.00 usec
PLW2        18.00000000 W
PLW12        0.28125000 W

F2 - Processing parameters
SI                65536
SF          125.8054215 MHz
WDW                  EM
SSB       0
LB                 1.00 Hz
GB        0
PC                 1.40
SR              -143.51 Hz
HZpPT          0.476837 Hz
```
