# Supplementary material for: Artificial Fluorescent Glucosinolates (F-GSLs) Are Transported by the Glucosinolate Transporters GTR1/2/3
Source: Int J Mol Sci. 2023 Jan 4;24(2):920. doi: 10.3390/ijms24020920 (PMC9862856; doi:10.3390/ijms24020920)
Supplement: Supplementary file 1 [file ijms-24-00920-s001.zip › NMR_rawdata_GSL-A_Bodipy_Rhodamine_Dansylamide.zip!/NMR raw data PK/GSL-A-Rhodamine/1/pdata/1/email_jic-210906-163907_oa_1_1.pdf]

no. of the sticker on the nmr tube 435
color of the label blue
workgroup klahn
KL-CJ4-006
protonpp.ibk_35 MeOD {D:\u} jic 23

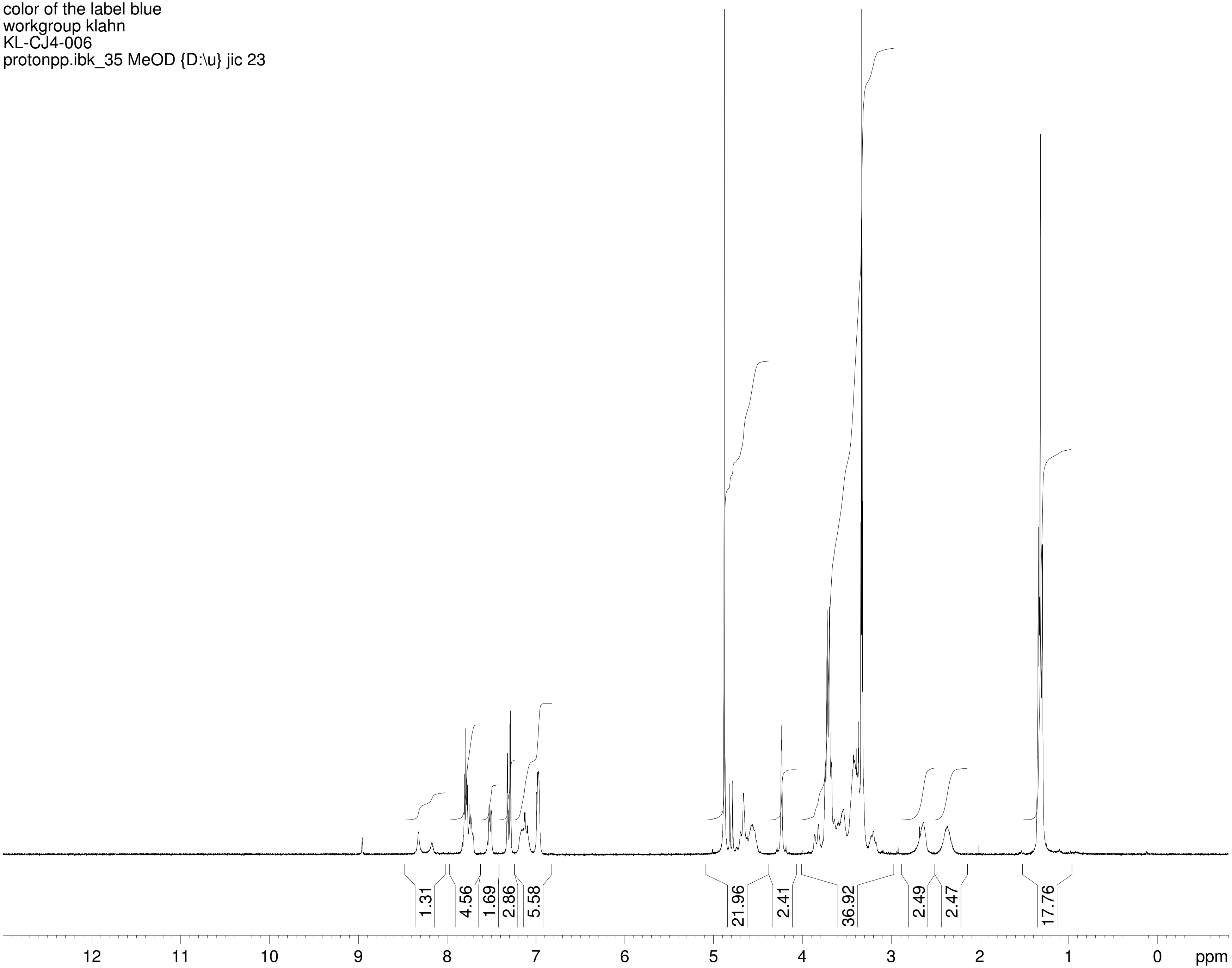

F1P: 13 ppm, F2P: -0.8 ppm

Scale: 0.6000 ppm/cm, 180.2 Hz/cm

Current Data Parameters

NAME	jic-210906-163907_oa
EXPNO	1
PROCNO	1

F2 - Acquisition Parameters

Date_	20210906
Time	21.11 h
INSTRUM	AVIIIHD300N
PROBHD	Z104275_0359 (
PULPROG	zg30
TD	49152
SOLVENT	MeOD
NS	24
DS	2
SWH	6203.474 Hz
FIDRES	0.252420 Hz
AQ	3.9616511 sec
RG	203.32
DW	80.600 usec
DE	6.50 usec
TE	298.0 K
D1	1.00000000 sec
TD0	1
SFO1	300.2618538 MHz
NUC1	1H
P0	4.00 usec
P1	12.00 usec
PLW1	10.85099983 W

F2 - Processing parameters

SI	32768
SF	300.2600000 MHz
WDW	EM
SSB	0
LB	0 Hz
GB	0
PC	1.00
