# Supplementary material for: Artificial Fluorescent Glucosinolates (F-GSLs) Are Transported by the Glucosinolate Transporters GTR1/2/3
Source: Int J Mol Sci. 2023 Jan 4;24(2):920. doi: 10.3390/ijms24020920 (PMC9862856; doi:10.3390/ijms24020920)
Supplement: Supplementary file 1 [file ijms-24-00920-s001.zip › NMR_rawdata_GSL-A_Bodipy_Rhodamine_Dansylamide.zip!/NMR raw data PK/GSL-A-Rhodamine/2/pdata/1/email_jic-210906-163907_oa_2_1.pdf]

KL-CJ4-006
c13cpd_l.ibk_35 MeOD {D:\u} jic 23

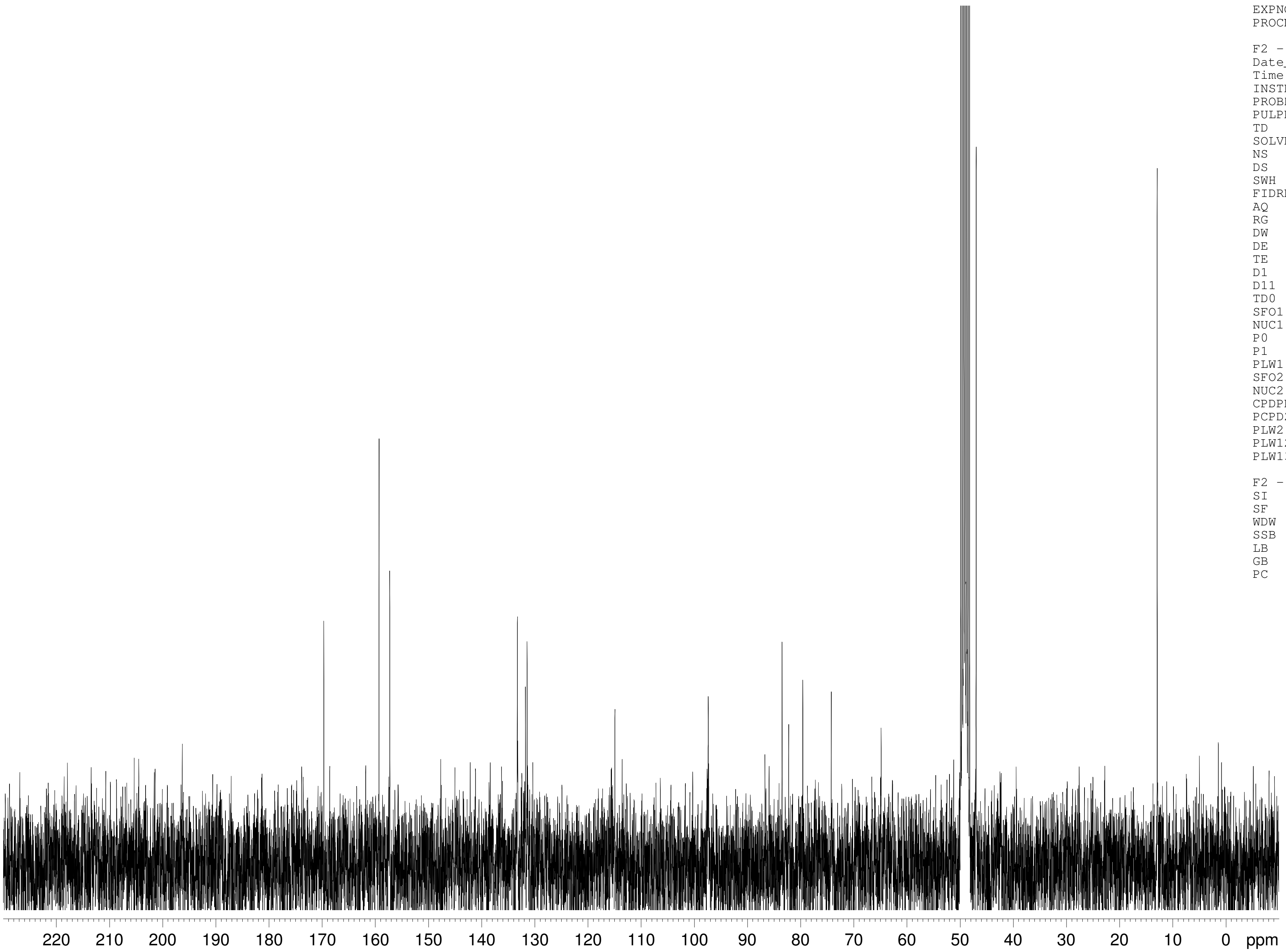

F1P: 230 ppm, F2P: -10 ppm

Scale: 10.00 ppm/cm, 755.0 Hz/cm

Current Data Parameters

NAME	jic-210906-163907_oa
EXPNO	2
PROCNO	1

F2 - Acquisition Parameters

Date_	20210906
Time	22.09 h
INSTRUM	AVIIIHD300N
PROBHD	Z104275_0359 (
PULPROG	zgpg30
TD	98304
SOLVENT	MeOD
NS	696
DS	4
SWH	19736.842 Hz
FIDRES	0.401547 Hz
AQ	2.4903679 sec
RG	32.64
DW	25.333 usec
DE	6.50 usec
TE	298.0 K
D1	2.00000000 sec
D11	0.03000000 sec
TD0	1
SFO1	75.5088171 MHz
NUC1	13C
P0	3.17 usec
P1	9.50 usec
PLW1	51.05799866 W
SFO2	300.2612010 MHz
NUC2	1H
CPDPRG[2	waltz16
PCPD2	90.00 usec
PLW2	10.85099983 W
PLW12	0.19291000 W
PLW13	0.09703100 W

F2 - Processing parameters

SI	65536
SF	75.5003304 MHz
WDW	EM
SSB	0
LB	1.00 Hz
GB	0
PC	1.40
