# Supplementary material for: Artificial Fluorescent Glucosinolates (F-GSLs) Are Transported by the Glucosinolate Transporters GTR1/2/3
Source: Int J Mol Sci. 2023 Jan 4;24(2):920. doi: 10.3390/ijms24020920 (PMC9862856; doi:10.3390/ijms24020920)
Supplement: Supplementary file 1 [file ijms-24-00920-s001.zip › NMR_rawdata_GSL-A_Bodipy_Rhodamine_Dansylamide.zip!/NMR raw data PK/GSL-A-Rhodamine/3/pdata/1/email_jic-210906-163907_oa_3_1.pdf]

KL-CJ4-006
c13dept135_l.ibk_35 MeOD {D:\u} jic 23

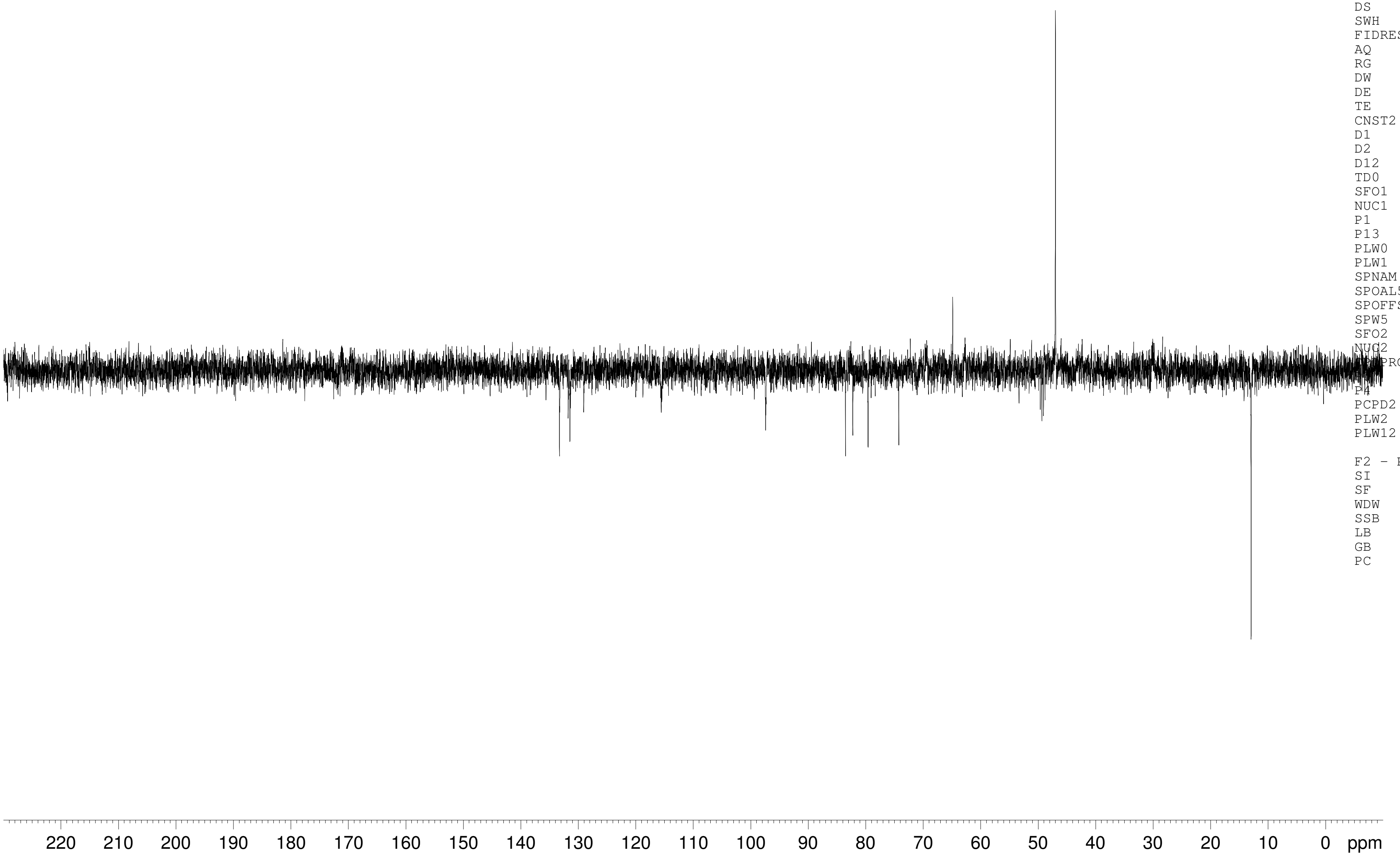

F1P: 230 ppm, F2P: -10 ppm

Scale: 10.00 ppm/cm, 755.0 Hz/cm

```
Current Data Parameters
NAME      jic-210906-163907_oa
EXPNO                        3
PROCNO                       1

F2 - Acquisition Parameters
Date_              20210906
Time                  22.36 h
INSTRUM         AVIIIHD300N
PROBHD     Z104275_0359 (
PULPROG          deptsp135
TD                    98304
SOLVENT                MeOD
NS                      344
DS                        4
SWH               19736.842 Hz
FIDRES             0.401547 Hz
AQ                2.4903679 sec
RG                   203.32
DW                   25.333 usec
DE                     6.50 usec
TE                    298.0 K
CNST2           145.0000000
D1               2.00000000 sec
D2               0.00344828 sec
D12              0.00002000 sec
TD0                       1
SFO1             75.5088171 MHz
NUC1                    13C
P1                     9.50 usec
P13                 2000.00 usec
PLW0       0 W
PLW1            51.05799866 W
SPNAM[5]       Crp60comp.4
SPOAL5                0.500
SPOFFS5    0 Hz
SPW5             7.04050016 W
SFO2            300.2612010 MHz
NUC2                     1H
[illegible]PRG[2            waltz16
[illegible]               12.00 usec
P4                    24.00 usec
PCPD2                 90.00 usec
PLW2            10.85099983 W
PLW12            0.19291000 W

F2 - Processing parameters
SI                    65536
SF               75.5003300 MHz
WDW                      EM
SSB       0
LB                     1.00 Hz
GB        0
PC                     1.40
```
