# Supplementary material for: Artificial Fluorescent Glucosinolates (F-GSLs) Are Transported by the Glucosinolate Transporters GTR1/2/3
Source: Int J Mol Sci. 2023 Jan 4;24(2):920. doi: 10.3390/ijms24020920 (PMC9862856; doi:10.3390/ijms24020920)
Supplement: Supplementary file 1 [file ijms-24-00920-s001.zip › NMR_rawdata_GSL-A_Bodipy_Rhodamine_Dansylamide.zip!/NMR raw data PK/Rhodamine/1/pdata/1/email_jic-210906-163744_oa_1_1.pdf]

no. of the sticker on the nmr tube 434
color of the label blue
workgroup klahn
KL-CJ4-004
protonpp.ibk_35 CDCl3 {D:\u} jic 22

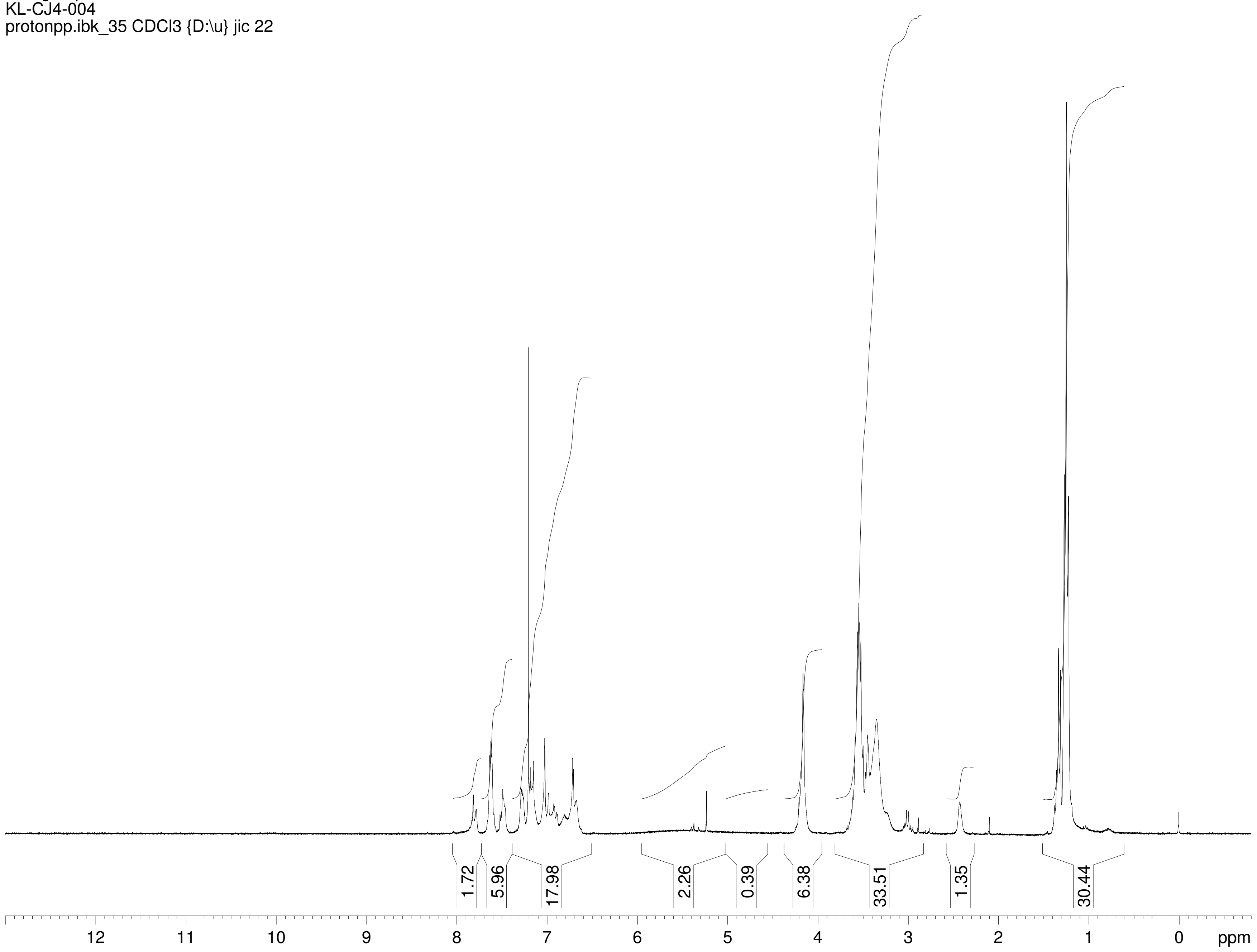

F1P: 13 ppm, F2P: -0.8 ppm

Scale: 0.6000 ppm/cm, 180.2 Hz/cm

```
Current Data Parameters
NAME     jic-210906-163744_oa
EXPNO                       1
PROCNO                      1

F2 - Acquisition Parameters
Date_            20210906
Time                19.34 h
INSTRUM       AVIIIHD300N
PROBHD   Z104275_0359 (
PULPROG              zg30
TD                  49152
SOLVENT             CDCl3
NS                     24
DS                      2
SWH              6203.474 Hz
FIDRES           0.252420 Hz
AQ              3.9616511 sec
RG                 203.32
DW                 80.600 usec
DE                   6.50 usec
TE                  298.0 K
D1             1.00000000 sec
TD0                     1
SFO1          300.2618538 MHz
NUC1                   1H
P0                   4.00 usec
P1                  12.00 usec
PLW1          10.85099983 W

F2 - Processing parameters
SI                  32768
SF            300.2600296 MHz
WDW                    EM
SSB      0
LB       0 Hz
GB       0
PC                   1.00
```
