# Supplementary material for: Artificial Fluorescent Glucosinolates (F-GSLs) Are Transported by the Glucosinolate Transporters GTR1/2/3
Source: Int J Mol Sci. 2023 Jan 4;24(2):920. doi: 10.3390/ijms24020920 (PMC9862856; doi:10.3390/ijms24020920)
Supplement: Supplementary file 1 [file ijms-24-00920-s001.zip › NMR_rawdata_GSL-A_Bodipy_Rhodamine_Dansylamide.zip!/NMR raw data PK/Rhodamine/2/pdata/1/email_jic-210906-163744_oa_2_1.pdf]

KL-CJ4-004
c13cpd_l.ibk_35 CDCl3 {D:\u} jic 22

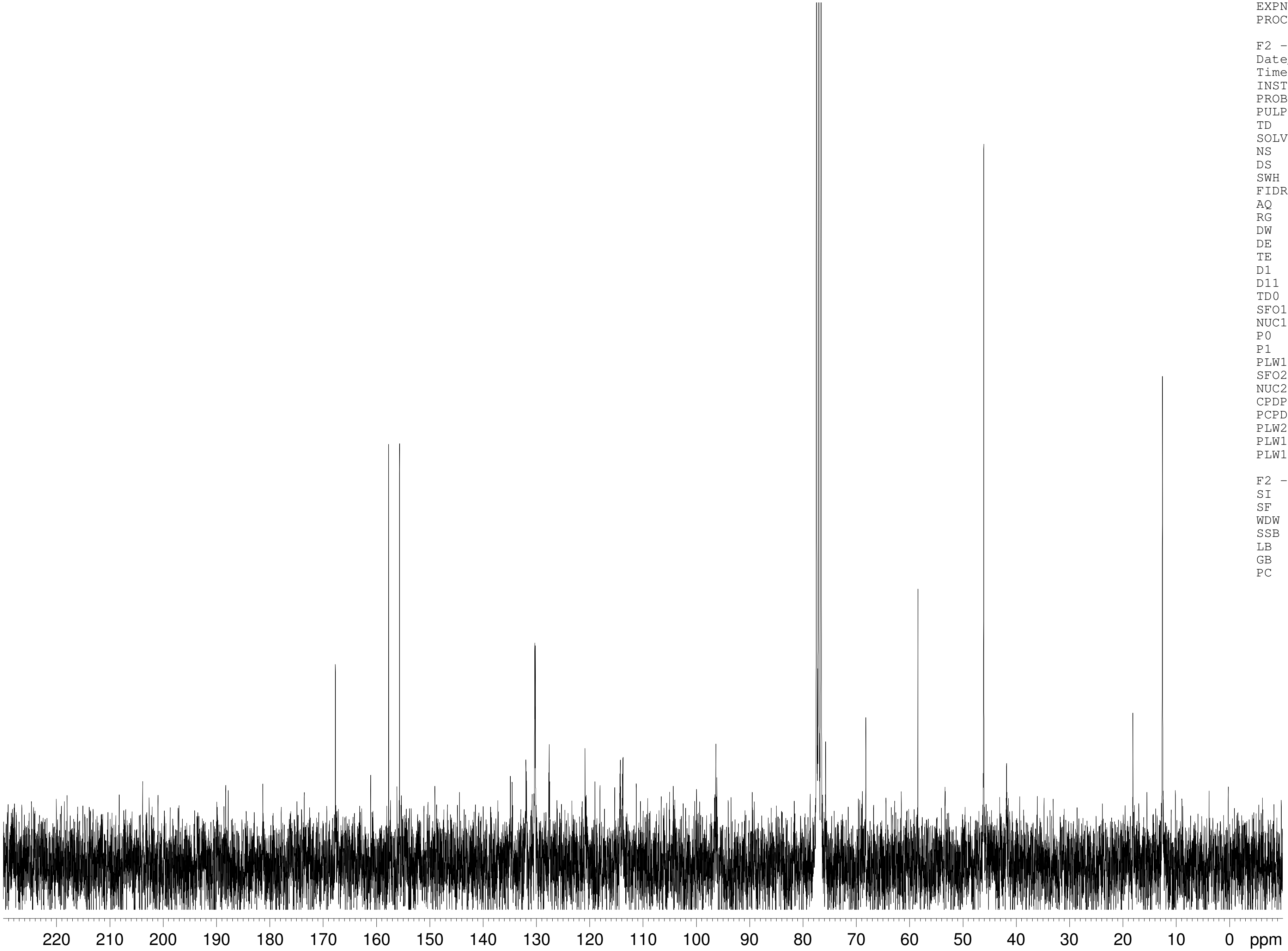


F1P: 230 ppm, F2P: -10 ppm

Scale: 10.00 ppm/cm, 755.0 Hz/cm

```
Current Data Parameters
NAME     jic-210906-163744_oa
EXPNO                       2
PROCNO                      1

F2 - Acquisition Parameters
Date_              20210906
Time                  20.29 h
INSTRUM         AVIIIHD300N
PROBHD   Z104275_0359 (
PULPROG              zgpg30
TD                    98304
SOLVENT               CDCl3
NS                      696
DS                        4
SWH              19736.842 Hz
FIDRES            0.401547 Hz
AQ               2.4903679 sec
RG                    46.97
DW                   25.333 usec
DE                     6.50 usec
TE                    298.0 K
D1              2.00000000 sec
D11             0.03000000 sec
TD0                       1
SFO1            75.5088171 MHz
NUC1                    13C
P0                     3.17 usec
P1                     9.50 usec
PLW1           51.05799866 W
SFO2           300.2612010 MHz
NUC2                     1H
CPDPRG[2            waltz16
PCPD2                 90.00 usec
PLW2           10.85099983 W
PLW12           0.19291000 W
PLW13           0.09703100 W

F2 - Processing parameters
SI                    65536
SF               75.5004420 MHz
WDW                      EM
SSB      0
LB                     1.00 Hz
GB       0
PC                     1.40
```
