# Supplementary material for: Artificial Fluorescent Glucosinolates (F-GSLs) Are Transported by the Glucosinolate Transporters GTR1/2/3
Source: Int J Mol Sci. 2023 Jan 4;24(2):920. doi: 10.3390/ijms24020920 (PMC9862856; doi:10.3390/ijms24020920)
Supplement: Supplementary file 1 [file ijms-24-00920-s001.zip › NMR_rawdata_GSL-A_Bodipy_Rhodamine_Dansylamide.zip!/NMR raw data PK/Rhodamine/3/pdata/1/email_jic-210906-163744_oa_3_1.pdf]

KL-CJ4-004
c13dept135_l.ibk_35 CDCl3 {D:\u} jic 22

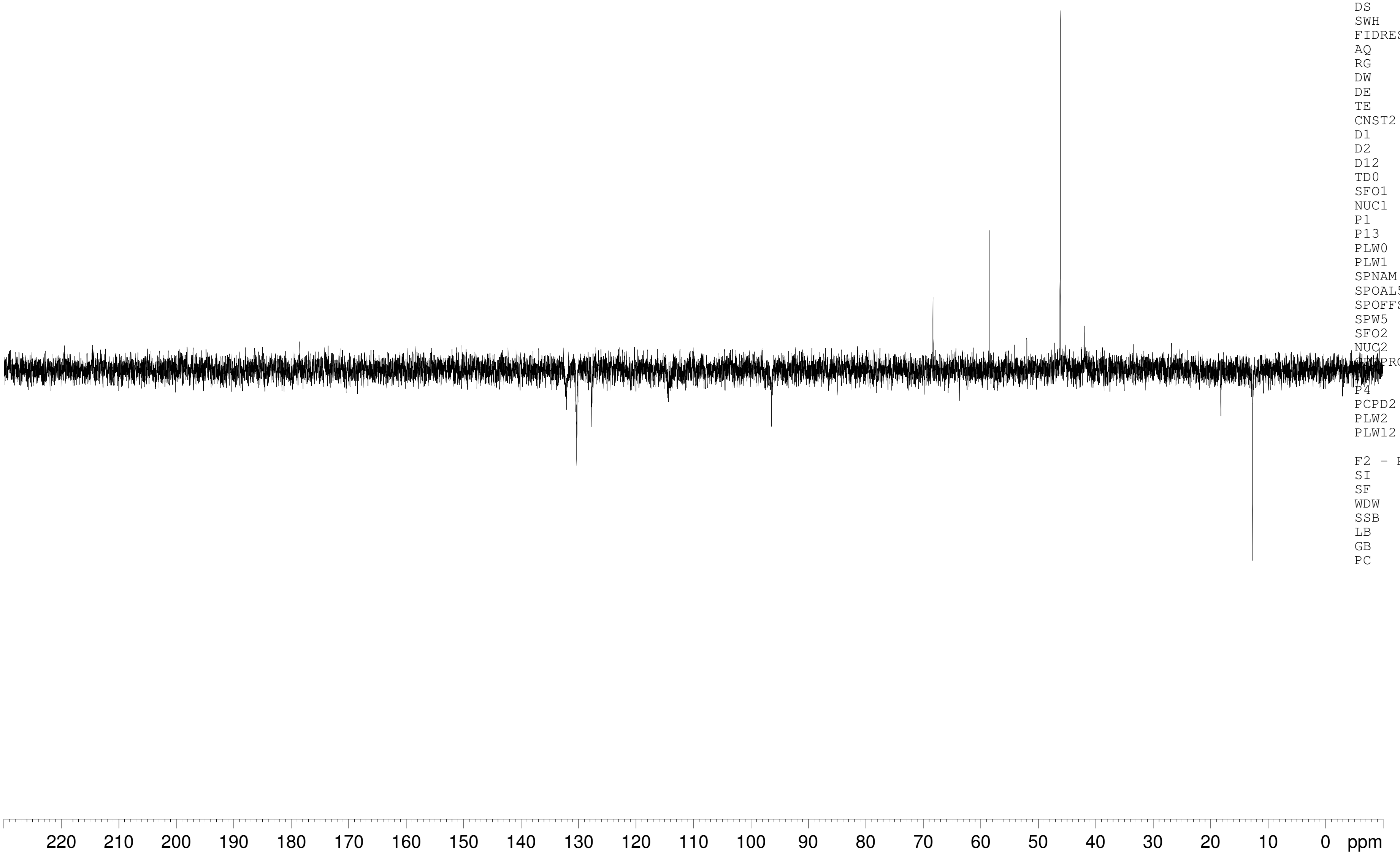

F1P: 230 ppm, F2P: -10 ppm

Scale: 10.00 ppm/cm, 755.0 Hz/cm

```
Current Data Parameters
NAME     jic-210906-163744_oa
EXPNO                  3
PROCNO                 1

F2 - Acquisition Parameters
Date_           20210906
Time               20.56 h
INSTRUM      AVIIIHD300N
PROBHD   Z104275_0359 (
PULPROG       deptsp135
TD                98304
SOLVENT           CDCl3
NS                  344
DS                    4
SWH           19736.842 Hz
FIDRES         0.401547 Hz
AQ            2.4903679 sec
RG               203.32
DW               25.333 usec
DE                 6.50 usec
TE                298.0 K
CNST2       145.0000000
D1           2.00000000 sec
D2           0.00344828 sec
D12          0.00002000 sec
TD0                   1
SFO1         75.5088171 MHz
NUC1                13C
P1                 9.50 usec
P13             2000.00 usec
PLW0     0 W
PLW1        51.05799866 W
SPNAM[5]    Crp60comp.4
SPOAL5            0.500
SPOFFS5  0 Hz
SPW5         7.04050016 W
SFO2        300.2612010 MHz
NUC2                 1H
CPDPRG[2        waltz16
[illegible]       12.00 usec
P4                24.00 usec
PCPD2             90.00 usec
PLW2        10.85099983 W
PLW12        0.19291000 W

F2 - Processing parameters
SI                65536
SF           75.5004356 MHz
WDW                  EM
SSB      0
LB                 1.00 Hz
GB       0
PC                 1.40
```
