# Supplementary material for: Artificial Fluorescent Glucosinolates (F-GSLs) Are Transported by the Glucosinolate Transporters GTR1/2/3
Source: Int J Mol Sci. 2023 Jan 4;24(2):920. doi: 10.3390/ijms24020920 (PMC9862856; doi:10.3390/ijms24020920)
Supplement: Supplementary file 1 [file ijms-24-00920-s001.zip › NMR_rawdata_GSL-A_Bodipy_Rhodamine_Dansylamide.zip!/NMR raw data PK/Rhodamine/4/pdata/1/email_jic-210906-163744_oa_4_1.pdf]

KL-CJ4-004
f19cpd.ibk_35 CDCl3 {D:\u} jic 22

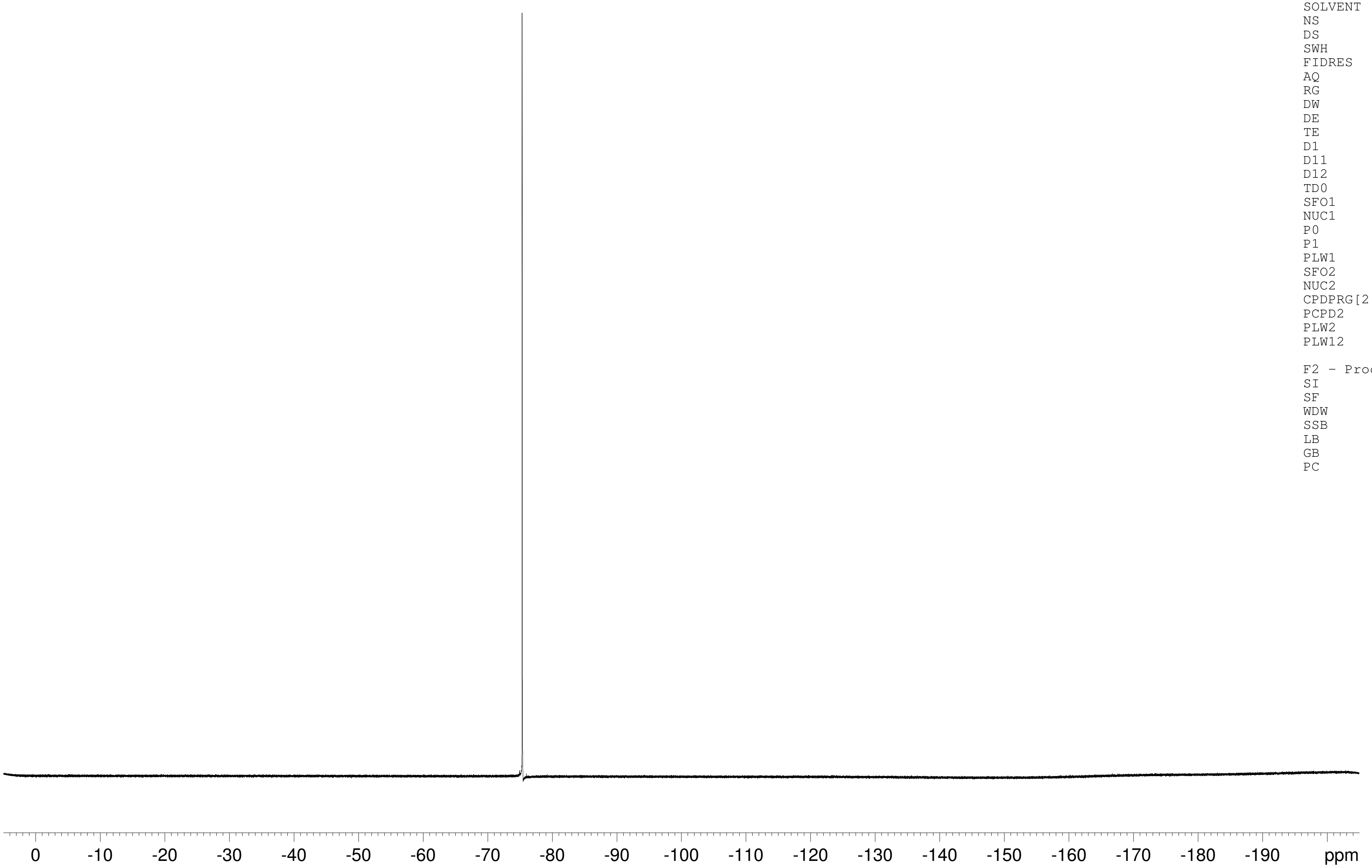

F1P: 5 ppm, F2P: -205 ppm

Scale: 8.750 ppm/cm, 2472 Hz/cm

```
Current Data Parameters
NAME      jic-210906-163744_oa
EXPNO                    4
PROCNO                   1

F2 - Acquisition Parameters
Date_             20210906
Time                 21.03 h
INSTRUM         AVIIIHD300N
PROBHD    Z104275_0359 (
PULPROG      zgfhigqn30.2
TD                  262144
SOLVENT              CDCl3
NS                      40
DS                       4
SWH              59523.809 Hz
FIDRES            0.454131 Hz
AQ               2.2020097 sec
RG                  203.32
DW                   8.400 usec
DE                    6.50 usec
TE                   298.0 K
D1              2.00000000 sec
D11             0.03000000 sec
D12             0.00002000 sec
TD0                      1
SFO1           282.4984248 MHz
NUC1                   19F
P0                    4.67 usec
P1                   14.00 usec
PLW1            8.62240028 W
SFO2           300.2612010 MHz
NUC2                    1H
CPDPRG[2           waltz16
PCPD2                90.00 usec
PLW2           10.85099983 W
PLW12           0.19291000 W

F2 - Processing parameters
SI                  131072
SF             282.5268000 MHz
WDW                     EM
SSB      0
LB                    0.30 Hz
GB       0
PC                    1.00
```
