# Supplementary material for: Artificial Fluorescent Glucosinolates (F-GSLs) Are Transported by the Glucosinolate Transporters GTR1/2/3
Source: Int J Mol Sci. 2023 Jan 4;24(2):920. doi: 10.3390/ijms24020920 (PMC9862856; doi:10.3390/ijms24020920)

^{1}H and ^{13}C NMR spectra of new compounds

GSL-A-Dansylamide

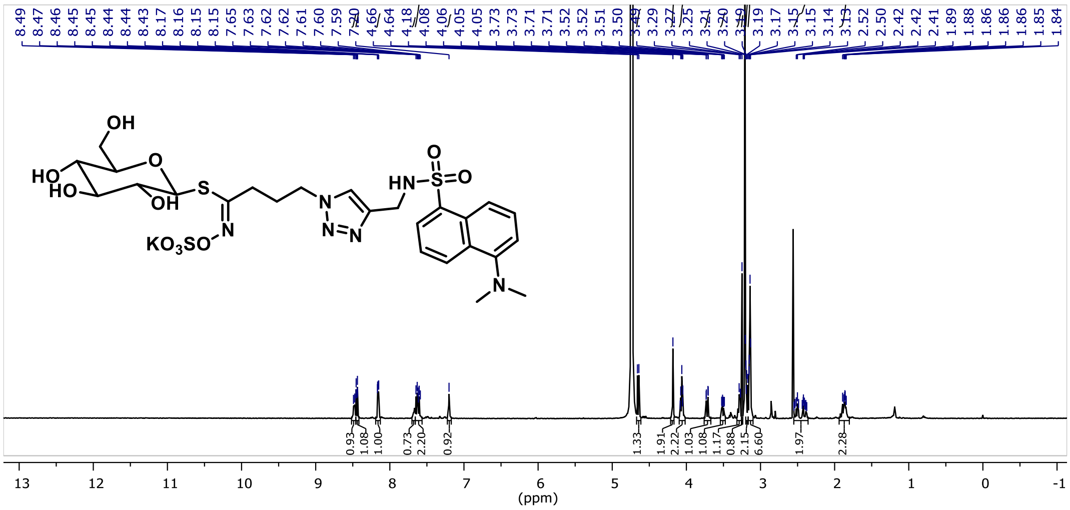

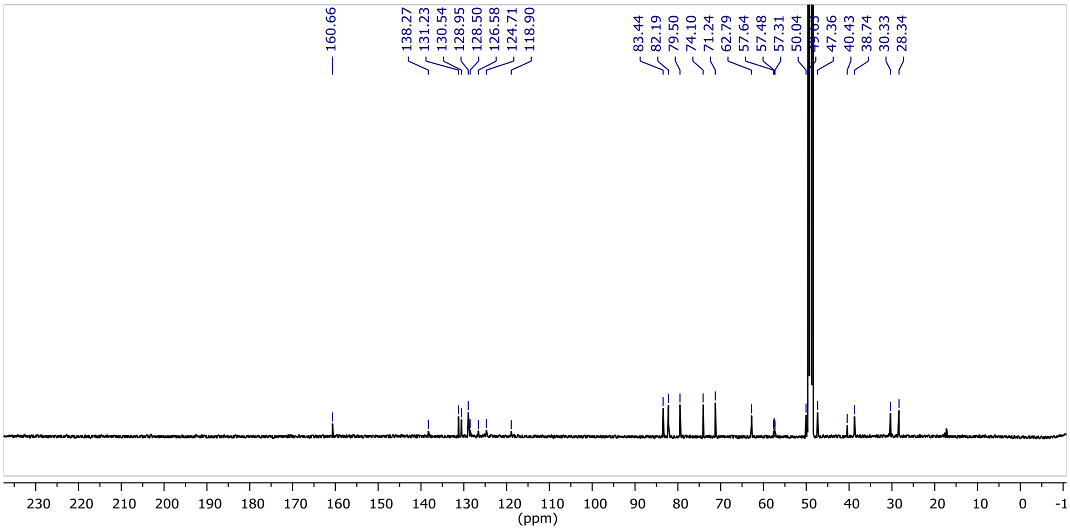

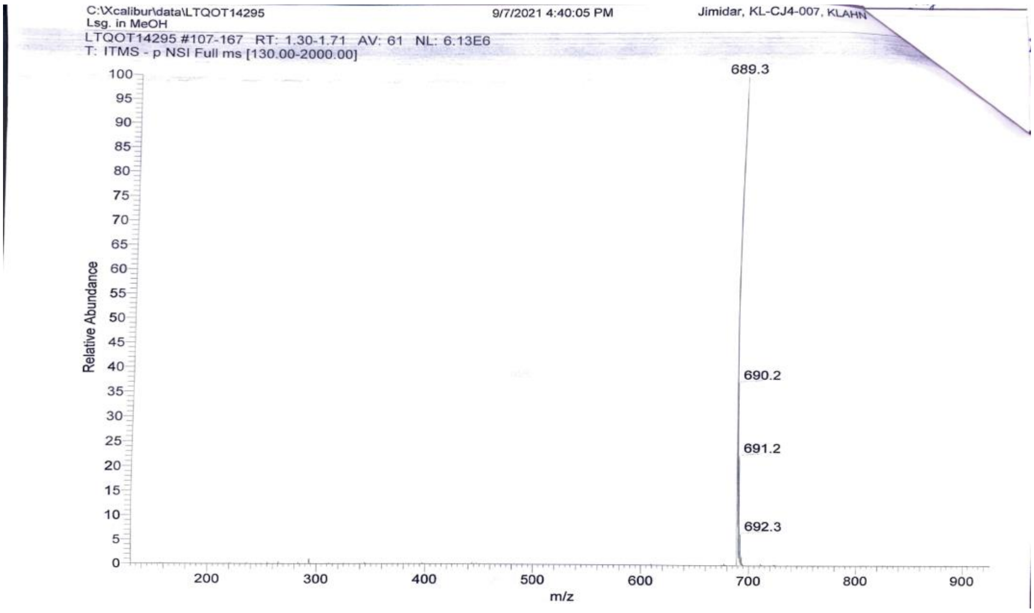

_N_-(6-(diethylamino)-9-(2-(4-(2-(prop-2-yn-1-yloxy)acetyl)piperazine-1-carbonyl)phenyl)-3_H_-xanthen-3-ylidene)-_N_-ethylethanaminium 2,2,2-trifluoroacetate - Rhodamine

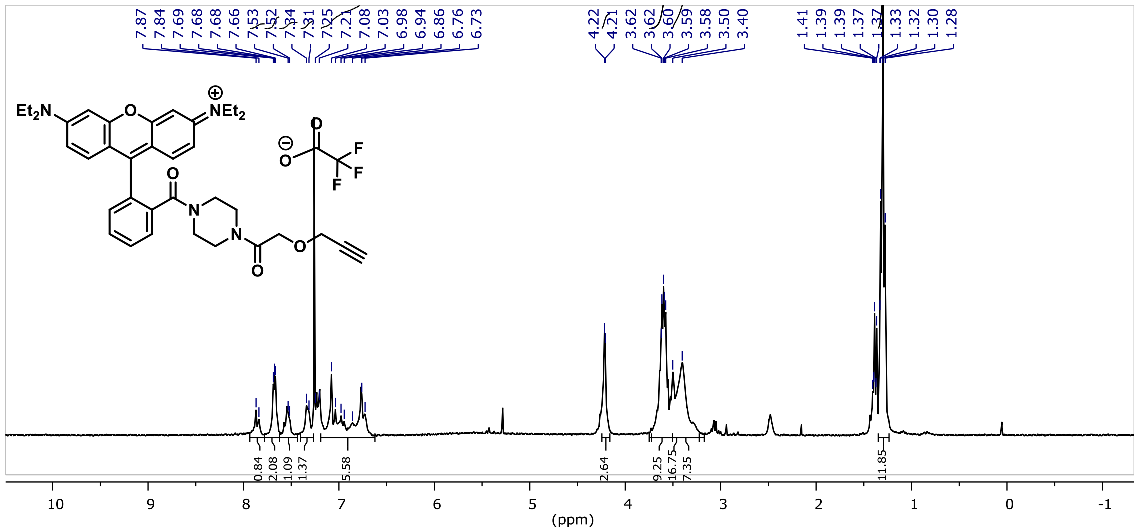

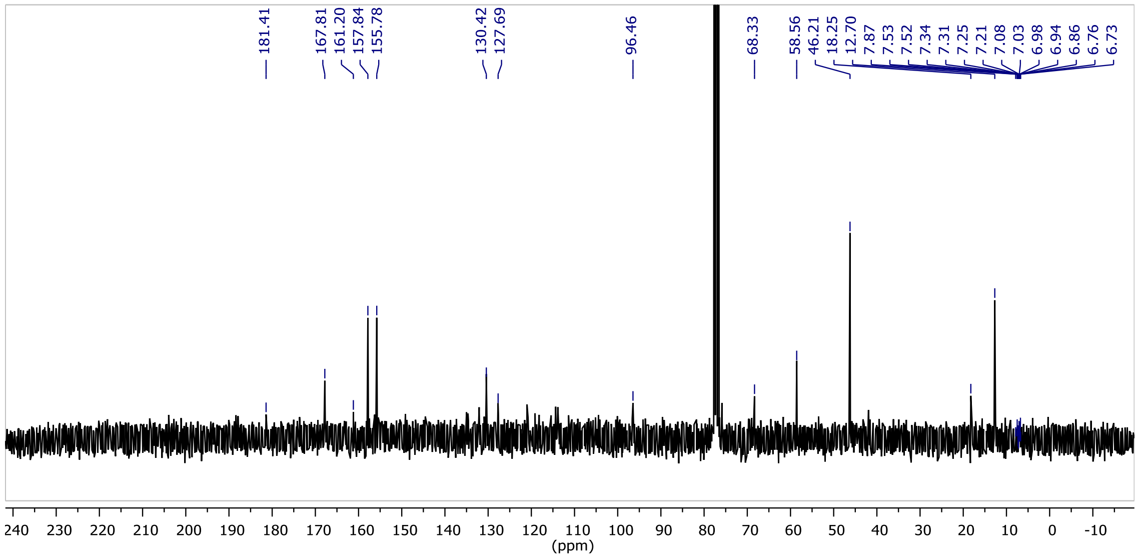

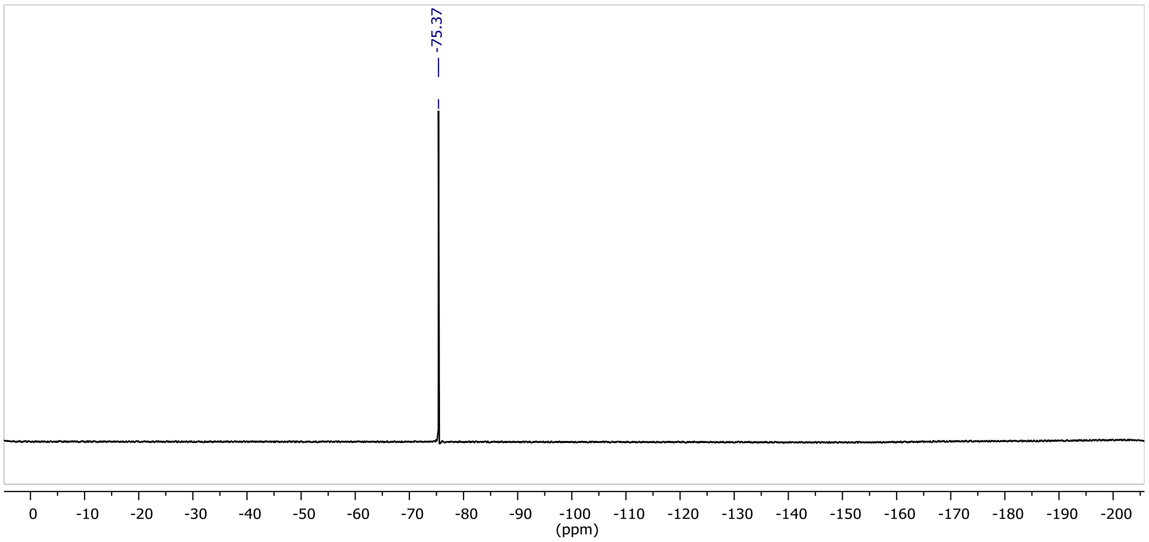

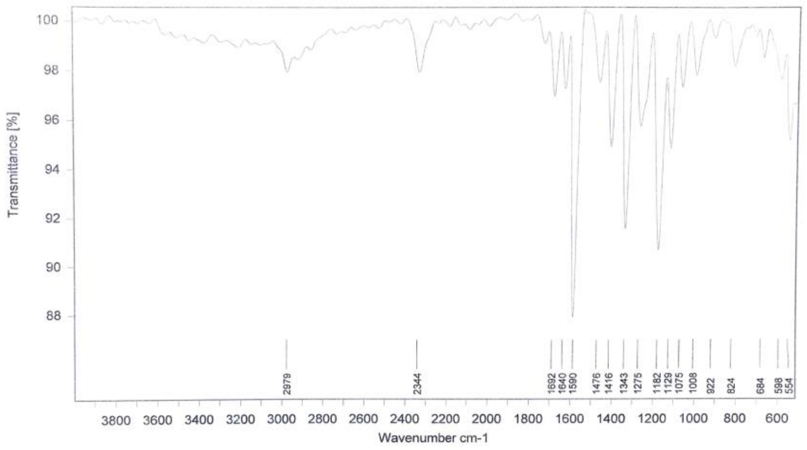

Instrument: Bruker Tensor 27	
Filename: jic30915.0	Number of Scans: 32
Sample Name: KL-CJ4-004	Operator Name: Default
Technique: Diamant-ATR	Date & Time of Measurement: 02.11.2021 15:44:00

02.11.2021

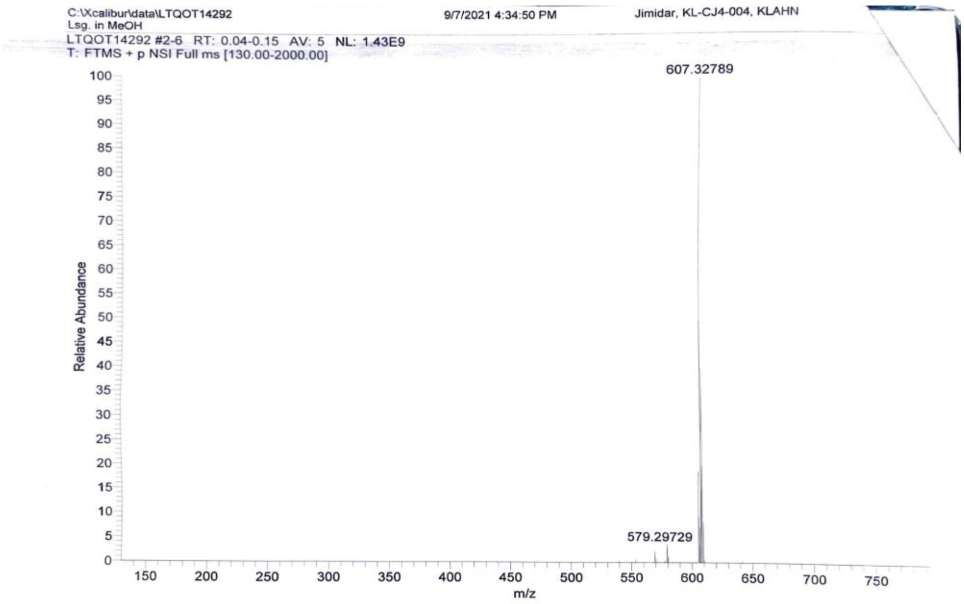

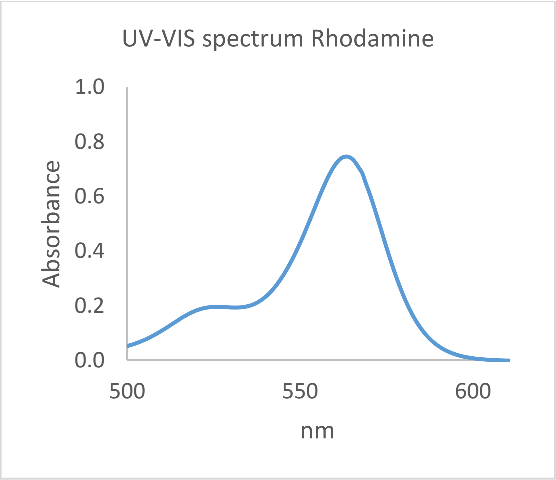

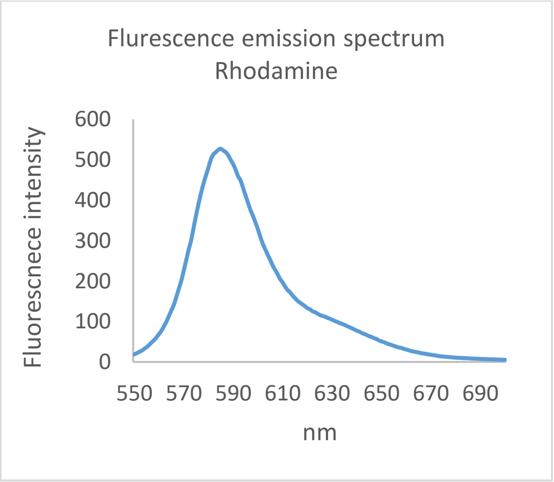

GSL-A-Rhodamine

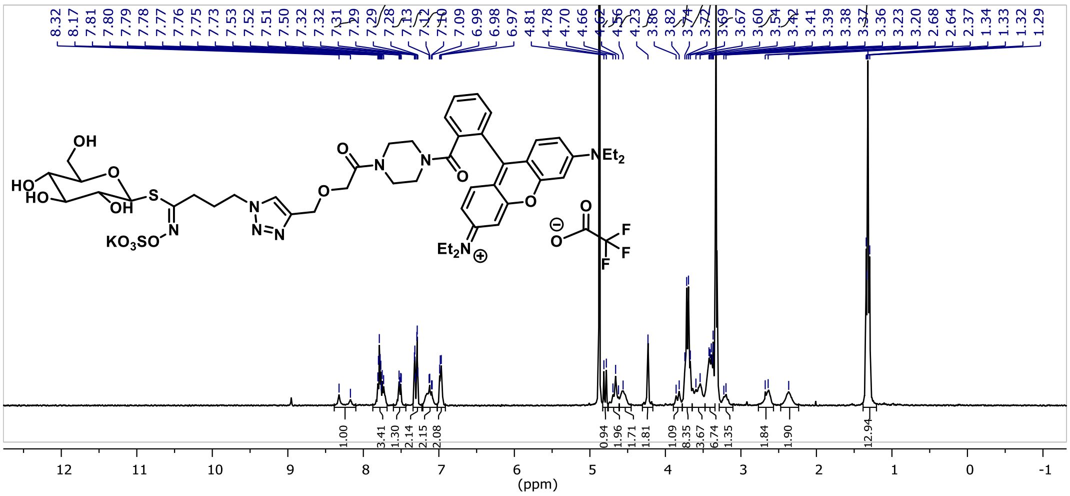

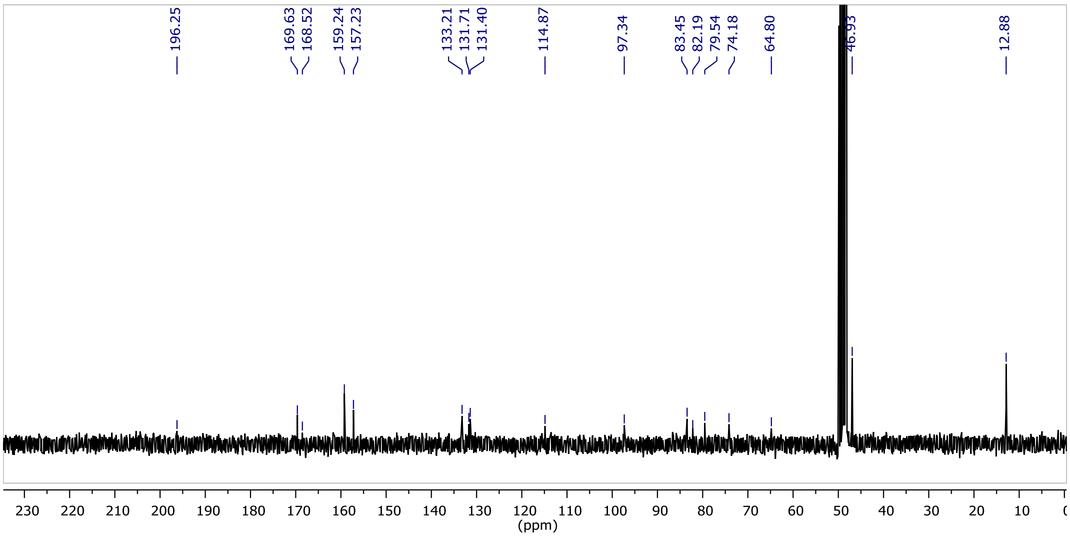

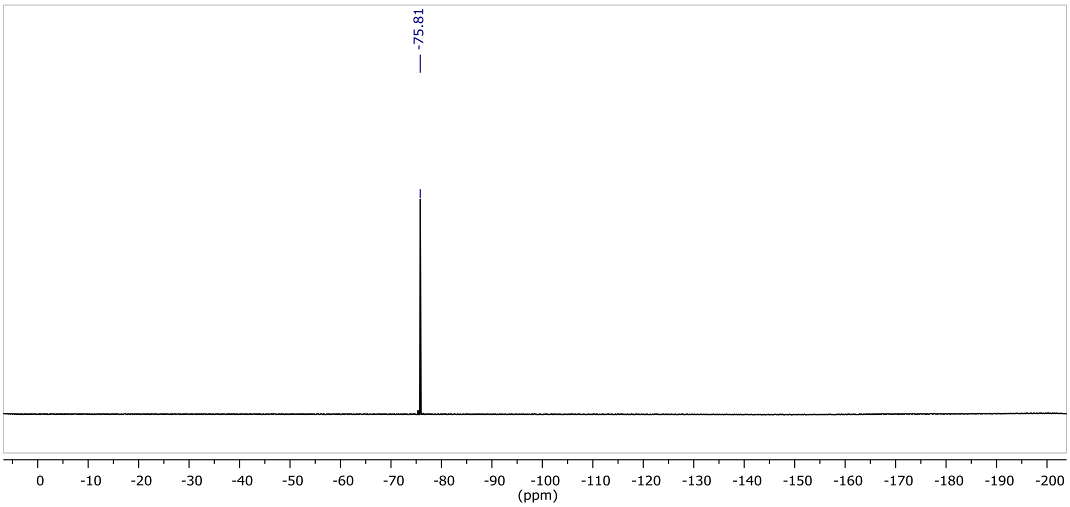

GSL-B-DNS - ^{1}H NMR (400 MHz, D_2O)

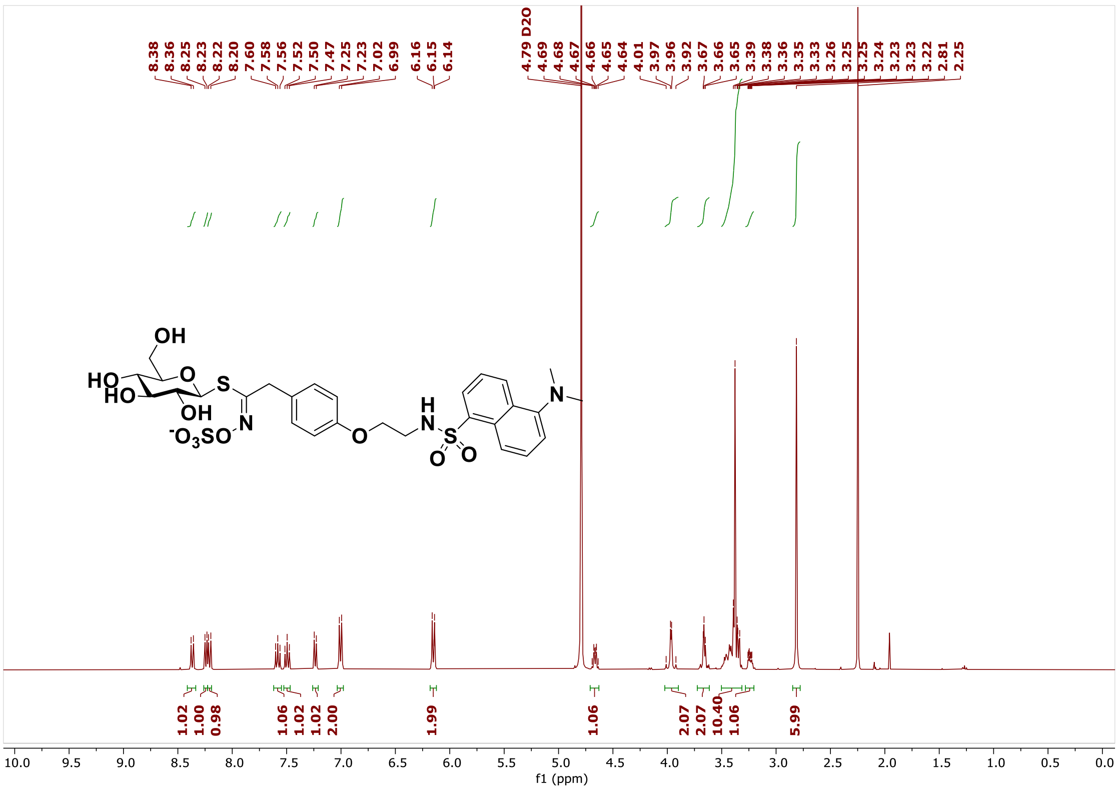

^{13}C NMR (100 MHz, D_2O, internal acetone)

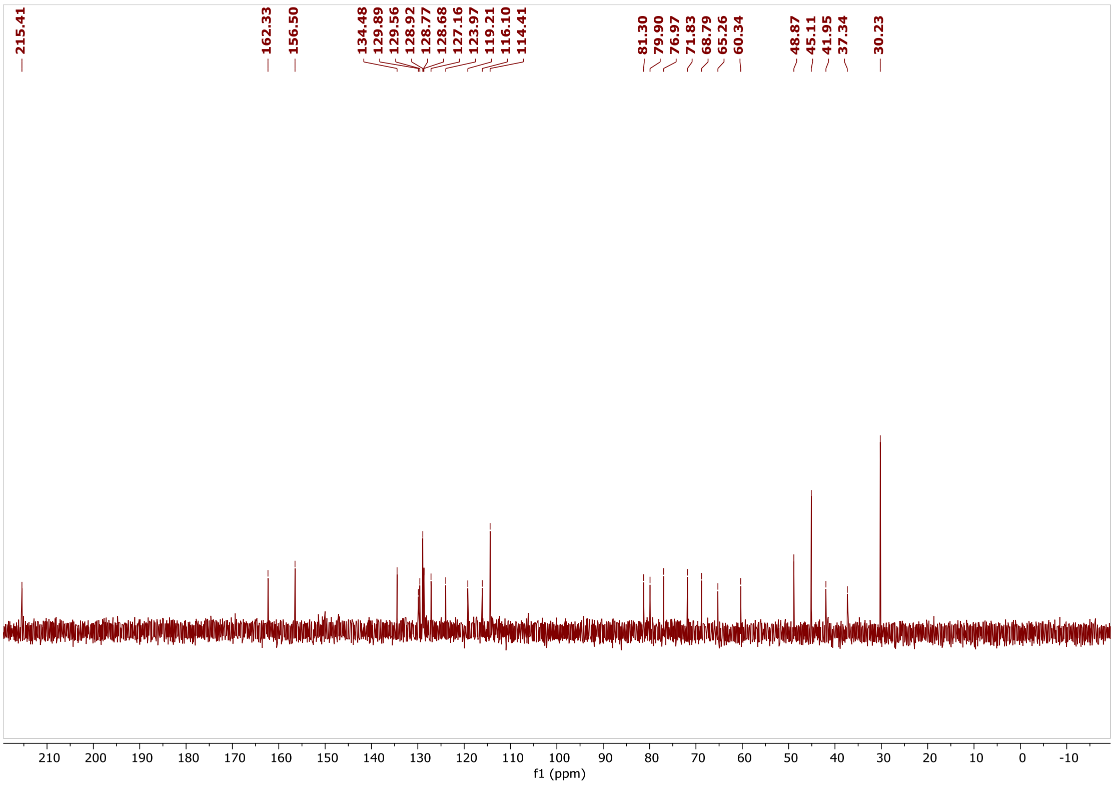

GSL-B-NBD - ^{1}H NMR (400 MHz, D_2O)

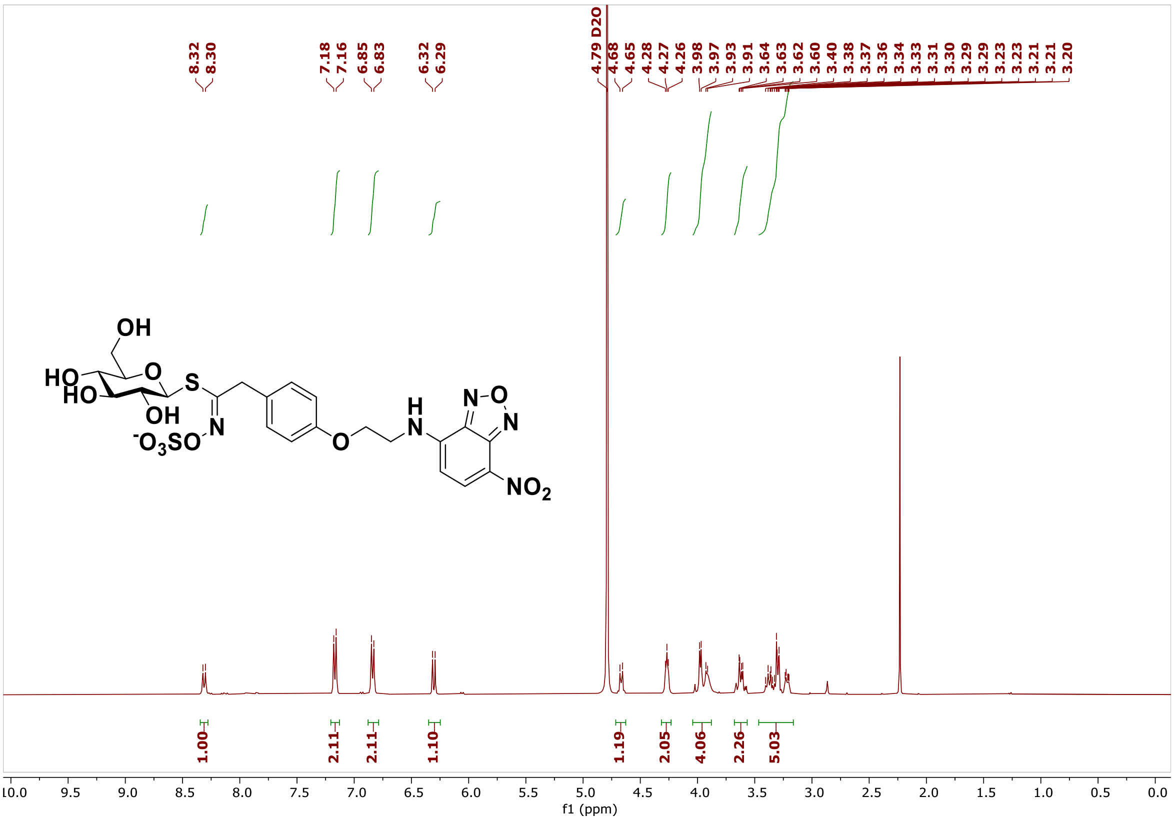

^{13}C NMR (100 MHz, D_2O, internal acetone)

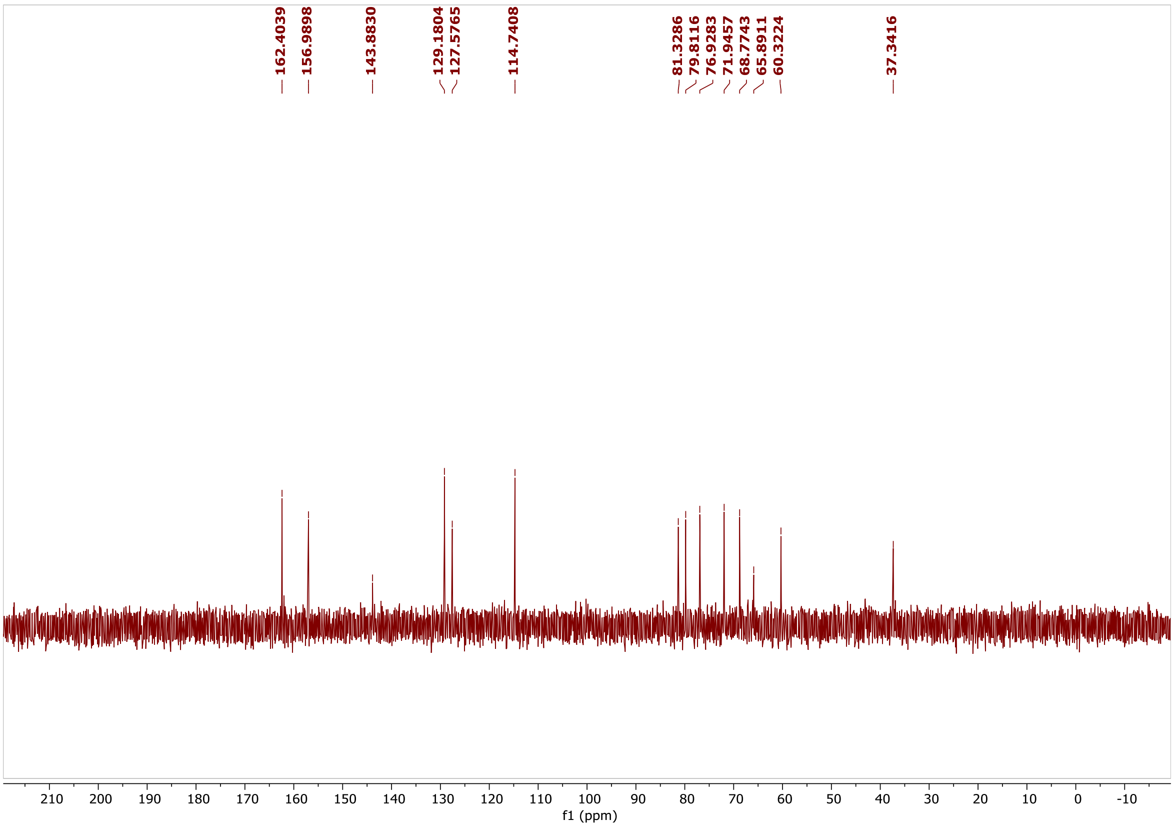

GSL-B-Fluorescein - ^{1}H NMR (400 MHz, CD_3OD)

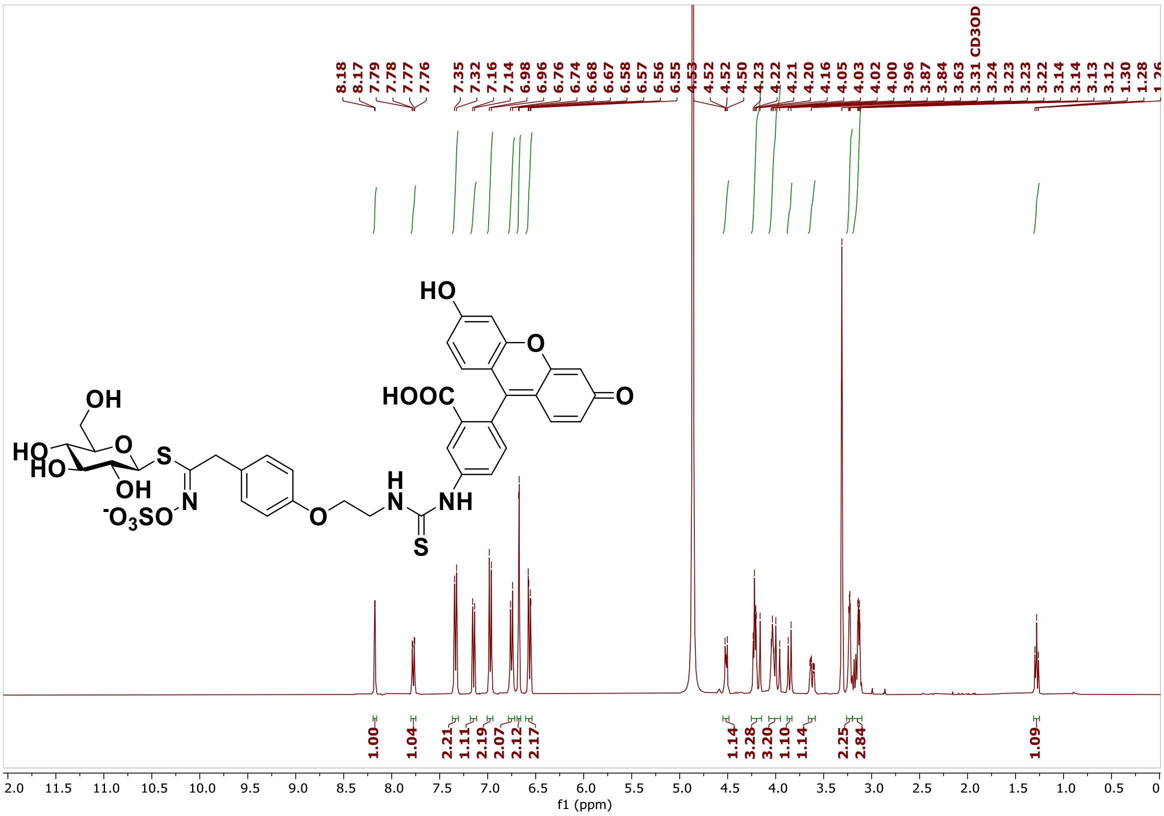

^{13}C NMR (100 MHz, CD_3OD)

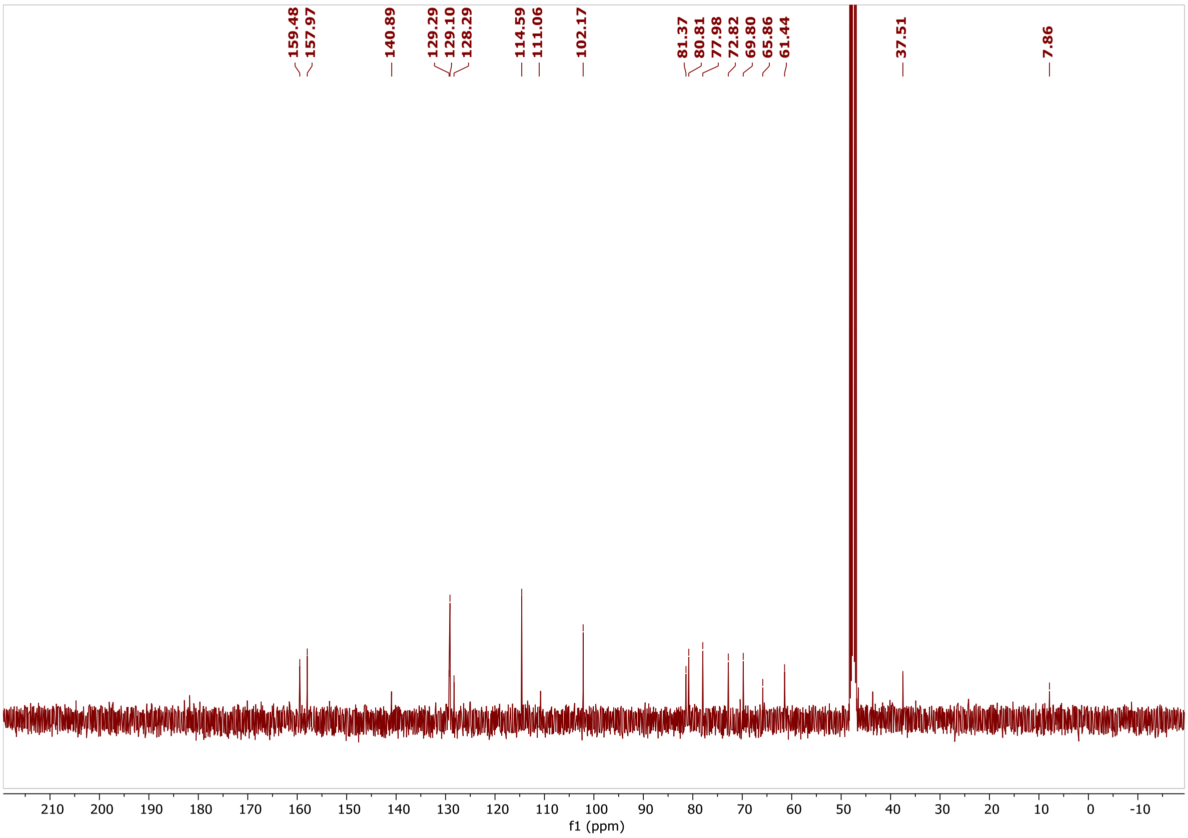

GSL-A-Fluorescein ^{1}H NMR (400 MHz, CD_3OD)

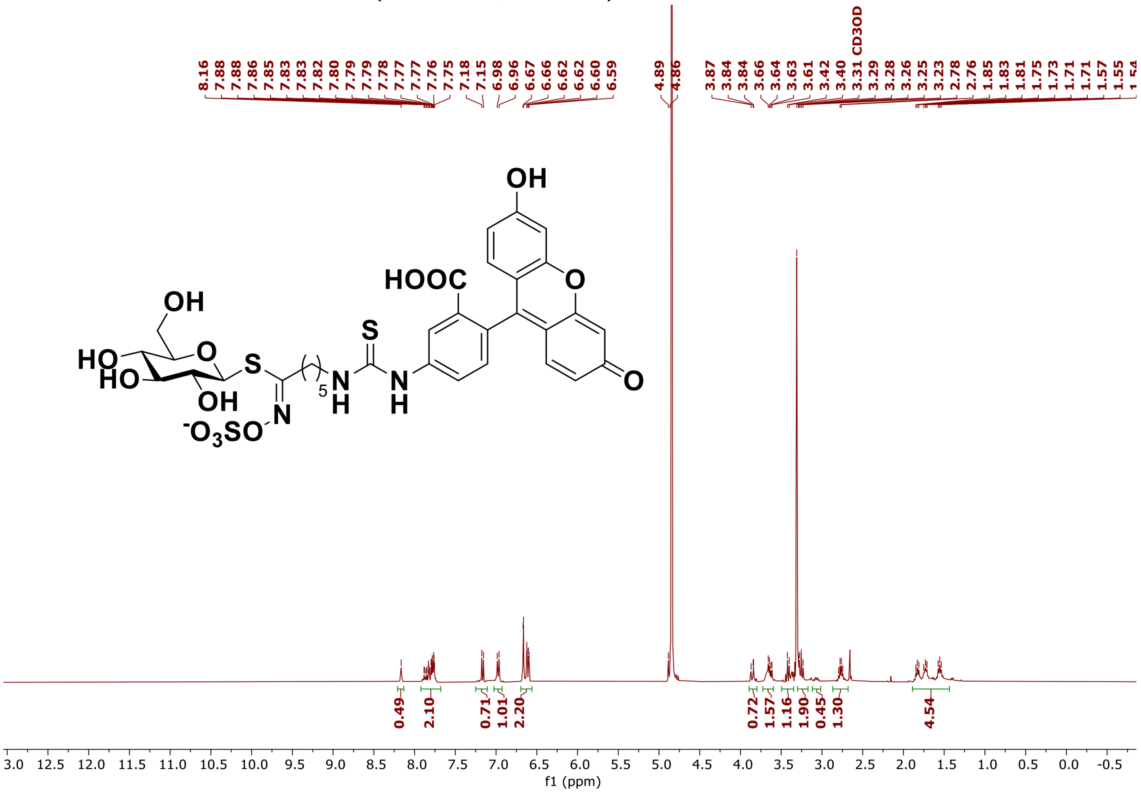

^{13}C NMR (100 MHz, CD_3OD)

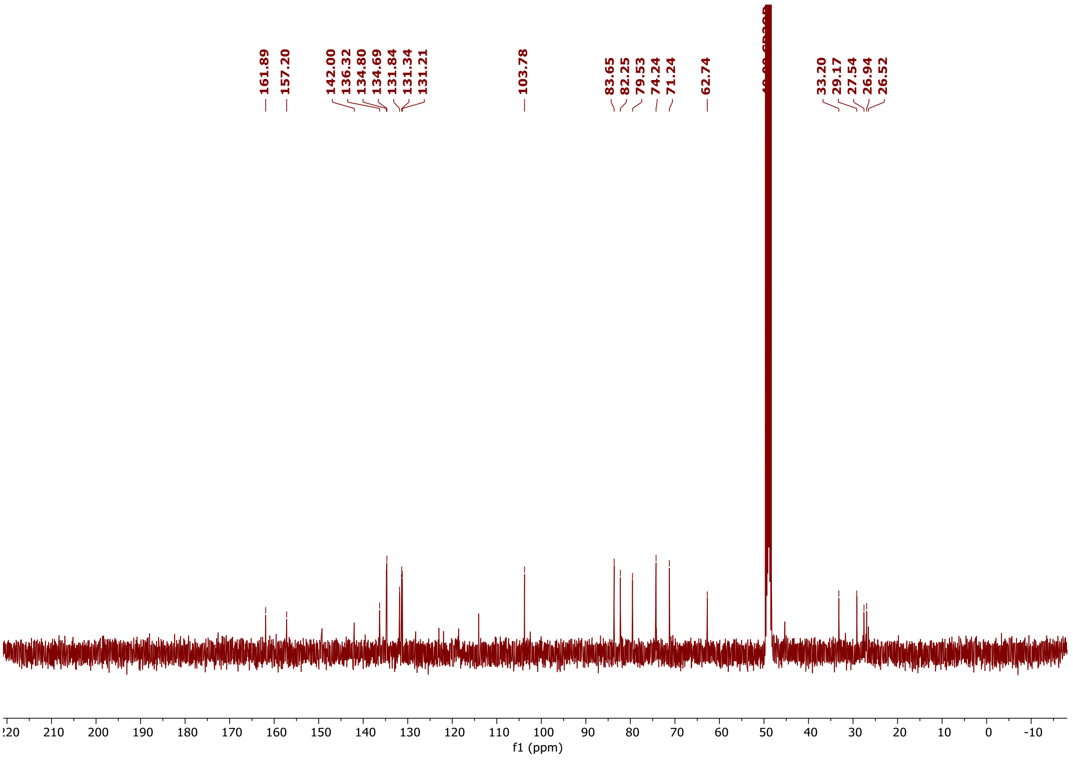

Supplement: Supplementary file 1 [file ijms-24-00920-s001.zip › NMR_spectra_SUPPLEMENTARY_Figurs.zip!/1H and 13C NMR spectra of new compounds.pdf]
